# Demographic and outcome differences in ICU patients with proven invasive candidiasis, possible invasive candidiasis and probable candida colonization: analysis of the EPIC II study population

**DOI:** 10.1186/cc9658

**Published:** 2011-03-11

**Authors:** D Kett, G Dimopoulos, E Azoulay, P Echeverria, JL Vincent

**Affiliations:** 1 University of Miami Miller School of Medicine, Miami, FL, USA; 2 University Hospital 'ATTIKON' Medical School, University of Athens, Greece; 3 Medical ICU, St-Louis Hospital and Paris VII University, Paris, France; 4 Erasme University Hospital, Brussels, Belgium

## Introduction

To evaluate differences in ICU patients with proven invasive candidiasis (Proven-IC), possible invasive candidiasis (Possible-IC), probable Candida colonization (colonized), and non-infected, noncolonized (non-infected) patients.

## Methods

EPIC II recruited 1,265 ICUs in 76 countries. Patient characteristics were collected on the study day. Outcome data were assessed at ICU and hospital discharge. Patients infected or colonized with non-Candida pathogens were excluded from this analysis. Patients with positive candida cultures may have had concurrent bacterial infections or colonization (*P < 0.05 compared with the non-infected group). Numerical values are reported as mean ± SD and length of stay (LOS) data as median (IQ).

## Results

A total of 13,796 adult patients were in a participating ICU on the study day. Of these, 110 had Proven-IC, 278 had Possible-IC, and 371 were colonized. In total, 6,507 patients were non-infected. Differences in patient characteristics and outcomes (Table 1) are reported.

**Table 1:** 

	Proven-IC	Possible-IC	Colonized	Non-infected
	(*n *= 110)	(*n *= 278)	(*n *= 371)	(*n *= 6,509)
SAPS II mean (SD)*	58 (14)	41 (15)	40 (16)	31 (14)
Mechanical ventilation (*n*, %)*	77 (71%)	204 (73%)	255 (70%)	2,822 (44%)
Vaspressors (*n*, %)*	37 (34%)	87 (31%)	129 (35%)	1,251 (19%)
ICU mortality (*n*, %)*	45 (42%)	93 (34%)	102 (29%)	649 (11%)
ICU LOS median (IQ)*	33 (18,52)	30 (16,52)	23 (11,41)	4 (1,4)

## Conclusions

ICU patients with proven invasive candidiasis, possible invasive candidiasis and candida colonization were more acutely ill and undergoing more ICU interventions than non-infected patients. The ICU mortality and LOS were also greater.
